# On the Use of the Purgative Neutral Salts in Cholera

**Published:** 1832-10

**Authors:** T. Ridgway


CHOLERA.

### *On the Use of the Purgative Neutral Salts in Cholera.*

By Dr. T. Ridgway.

It was among the first points of instruction I remember to have received in the practice of medicine, more than thirty years since, to administer, in those affections of the intestinal canal that are marked by pain and frequent dejections, the sulphate of soda, or Glauber's salt, in the full dose of an ounce, simply dissolved in water, as the remedy by which they would be most effectually and promptly terminated; and, accordingly, such I found to be the result. I have consequently adhered to this method; and, when the present epidemic first shewed itself, in August last year, I did not hesitate to resort to this admirable medicine as usual, and with the same manifest advantage, and I even repeatedly represented its usefulness in anticipation of the appearance of the more aggravated form of the disease.

With this experience before me, it was natural I should feel surprised at the denunciation which, when this unhappy event did actually take place, was specially directed against this class of remedies; and the more so, when I saw the very insufficient grounds on which the opinion against their use was founded.

I thought it exceedingly unfortunate that, on the accidental result in a single instance, and the unsound and superficial belief that a medicine possessing evacuant properties must necessarily be injurious in every state of augmentation of the intestinal discharges, the purgative neutral salts, fraught as they are with excellent properties, should have been so peremptorily rejected; and I therefore, in those opportunities that have been afforded me, repudiated the objections thus unjustly made against them.

The application of the term diarrhœa to certain stages of this epidemic, is not only obviously improper, but has had a direct and fatal influence on the treatment, by perverting the judgment and leading to erroneous inferences, and to the adoption of means that are injudicious and of a hurtful tendency. That of Erythematic Enteritis, which has been applied to similar affections by medical writers, while it corresponds with the appearances, seems less liable to mislead, and may be equally employed to designate the disease in all its forms and stages.

It seems probable that the morbid principle which is the cause of this disease, wheresoever generated or however transmitted, being received by the mouth, passes, in consequence of the incessant act of deglutition, with the saliva into the stomach, and onwards to the intestines, producing its effect on the mucous membrane of these organs, and thence extending its influence over the whole system. Upon this ground, should such remedies as the saline purgatives obtain a preference, which, while they act in an especial manner on the muco-intestinal expansion, pass, and are carried with their effects, into the extremest limits of the circulation.

By means of the saline purgatives, frequently alone, sometimes preceded by the tartar emetic, *en lavage*, as it is termed, and occasionally followed by powders of the nitrate of potass, with the tartar emetic, I have treated nearly sixty persons affected with the present epidemic, seldom with any protraction of the disease, never with a fatal result.

This method of treatment is declared, in the report from Paris, to have been eminently successful; and it is stated to have been acquired from the Germans, but I believe it to be nearly that which was long employed in this country, until superseded by the innovations of the present day.

I have meant these observations to apply chiefly to the milder form of the disease; for such, in comparison with what has been shewn me as the malignant or true cholera, have, in the greater number of instances, been those cases that have come under my care. But if, as asserted by some, they are identical, and differ only in degree, I may insist with confidence on this treatment, as applicable to all, by removing rapidly the incipient stages, and thereby obviating the consequent developement of dangerous symptoms: or, if they are really different, yet that analogy which permits to doubt of their identity would also afford reasonable grounds for the inference, that the means that are effectual in repressing the disease in one of its forms will be proportionately beneficial in the other.

But the phantom of debility (supported sometimes, it is true, by appearances,) seems to have obtained such absolute possession of the minds of all, that opiates, and excitants, and astringents, are flown to under every circumstance of the disease; and a want of success seems not to be admitted as any proof of their inutility. It is only to dare to be emancipated from this thraldom, and every difficulty will vanish. Success is surely a criterion of the usefulness of any method, and a want of it the contrary; and has it not, upon this principle, been fatally proved that such remedies as have been mentioned are followed by baleful results: is it not, therefore, an obvious consequence, that the reverse of these will exert a salutary influence? A consequence it would be a rejection of the principles of common sense to refuse to adopt.

To me, what has been passing concerning the use of saline medicines in this disease, and in febrile affections generally, is indeed incomprehensible; for, from what I have seen of the practice of medicine, it has seemed to me to be a means of cure that formerly, at least, obtained almost universally, and which continues, in a greater or less degree, to pervade the practice even of those who are most imbued with the prevailing doctrines.

When the theories which introduced these remedies to general use fell into neglect, they remained unsustained otherwise than by their intrinsic advantages; and their mode of operation necessarily became undefined and obscure, so that every one has felt himself at liberty to make what application of them he has thought proper; and it is one of their peculiar advantages that they can seldom be misapplied.

If cholera is to be prevented by medicine, I think it probable that sulphur may be found effectual for this purpose; and this formula, which I have taken from Boerhaave's Consultations, and found useful on other occasions, may be a convenient mode of administering it: ℞. Sulphur. loti. ℥iij.; P. Myrrhæ opt. ℥iss.; Pulv. Rhei opt. ℥i.; Terebinth. venet. ℥i. Let these ingredients be rubbed well together, and then, with the addition of a little water, formed into a mass, to be divided into three-grain pills, of which two or more may be taken in the morning or evening, or oftener.

The sulphur bath, also, by its general influence on the skin and system at large, as well as that, for some time after its application, it involves the whole person in a sulphureous atmosphere, promises to become an excellent means of protection against this formidable disease.

*London; Sept 10th,* 1832.

